# CCR5/CXCR4 Dual Antagonism for the Improvement of HIV Infection Therapy

**DOI:** 10.3390/molecules24030550

**Published:** 2019-02-02

**Authors:** Fedora Grande, Maria Antonietta Occhiuzzi, Bruno Rizzuti, Giuseppina Ioele, Michele De Luca, Paola Tucci, Valentina Svicher, Stefano Aquaro, Antonio Garofalo

**Affiliations:** 1 Department of Pharmacy, Health and Nutritional Sciences, University of Calabria, Ampliamento Polifunzionale, Via P. Bucci, 87036 Rende (CS), Italy; mariaantonietta.occhiuzzi@unical.it (M.A.O.); giuseppina.ioele@unical.it (G.I.); michele.deluca@unical.it (M.D.L.); paola.tucci@unical.it (P.T.); stefano.aquaro@unical.it (S.A.); antonio.garofalo@unical.it (A.G.); 2 CNR-NANOTEC, Licryl-UOS Cosenza and CEMIF.Cal, Department of Physics, University of Calabria, Via P. Bucci, 87036 Rende (CS), Italy; 3 Department of Experimental Medicine, University of Rome “Tor Vergata”, 00133 Rome, Italy; valentina.svicher@uniroma2.it

**Keywords:** chemokine receptors, dual drugs, HIV entry, AIDS

## Abstract

HIV entry in the host cell requires the interaction with the CD4 membrane receptor, and depends on the activation of one or both co-receptors CCR5 and CXCR4. Former selective co-receptor antagonists, acting at early stages of infection, are able to impair the receptor functions, preventing the viral spread toward AIDS. Due to the capability of HIV to develop resistance by switching from CCR5 to CXCR4, dual co-receptor antagonists could represent the next generation of AIDS prophylaxis drugs. We herein present a survey on relevant results published in the last few years on compounds acting simultaneously on both co-receptors, potentially useful as preventing agents or in combination with classical anti-retroviral drugs based therapy.

## 1. Introduction

The most successful treatment of patients infected with HIV-1 (human immunodeficiency virus 1) resides so far in the administration of a combination of antiretroviral agents targeting specific proteins necessary to the viral replication. Such a therapeutic strategy, known as highly active antiretroviral therapy (HAART) [1,2], has shown to be particularly effective during the progression of the full-blown viral infection, whereas the same approach was less applicable in the early stages of the infection when infected people are not warned by specific symptoms. The most common agents included in HAART are inhibitors of viral entry and fusion, or drugs targeting reverse transcriptase, integrase, and protease enzymes. An alternative strategic approach focuses on a multi-target inhibition of the viral entry, before a full spreading of the virus into host cells. The viral entry consists of a complex sequence of events mediated by several viral/cellular membrane proteins, with the interaction between the viral glycoprotein gp120 and CD4 cellular receptor constituting an essential initial step. A further interaction of such glycoprotein with any of the two co-receptors CC-chemokine receptor 5 (CCR5) and CXC-chemokine receptor 4 (CXCR4) is also needed to promote appropriate conformational modifications during such an early stage of the viral cycle [3].

**Figure 1:**
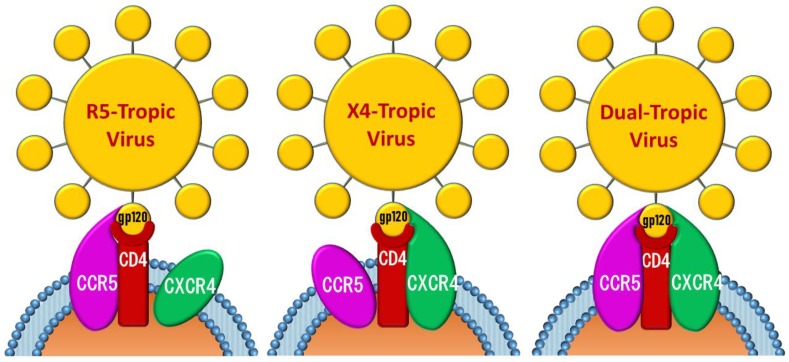
Schematic representation of chemokine receptors mediating HIV cells entry.

Both CCR5 and CXCR4 are G-protein coupled chemokine receptors determining HIV-1 cellular tropism. In particular, viruses preferentially utilizing CCR5 (R5 strains) are responsible for the primary cell infection, being this co-receptor mainly expressed on macrophages. On the other hand, the viral replication capacity is attributed to viruses preferentially interacting with CXCR4 (X4 strains), being this second co-receptor mainly expressed on T-lymphocytes. Finally, dual-tropic viruses are defined as those using both co-receptors (Figure 1) [4,5].

**Figure 2:**
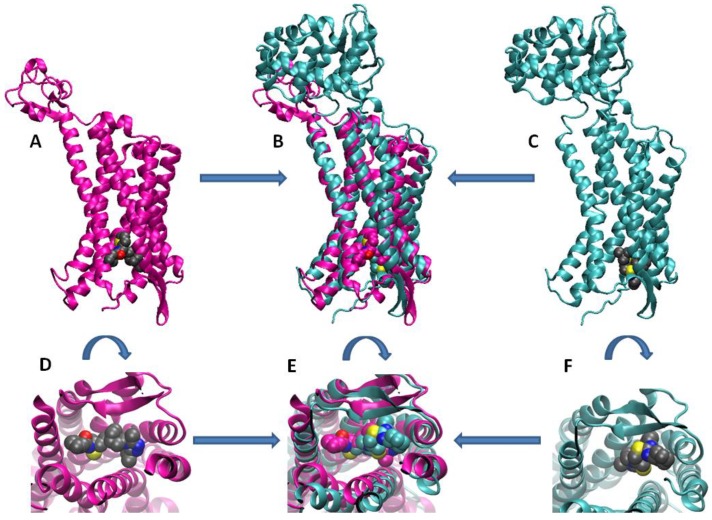
Similarities in the overall structures and binding pockets of CCR5 and CXCR4. Structures of complexes are taken from the Protein Data Bank entry 6AKX (magenta) [9] and 3ODU (cyan) [10] for CCR5 and CXCR4, respectively, and superimposed by least squares fit on protein Cα atoms. (Top) Overall structure of (**A**) CCR5, (**B**) both receptors, and (**C**) CXCR4; (bottom) detail of the binding site of (**D**) CCR5, (**E**) both receptors, and (**F**) CXCR4. The ligands bound to CCR5 and CXCR4 (the 1-heteroaryl-1,3-propanediamine derivative A4R and the antagonist IT1t, respectively) are shown with atoms in standard colours (C, grey; N, blue; O, red; S, yellow) in panels A, C, D and F, and with C atoms in the same colour as their receptor (magenta for A4R, and cyan for IT1t) in panels B and E.

Whereas a large number of agents acting selectively on either CCR5 or CXCR4 have been described in the literature [6,7], only a few dual antagonists have been so far identified, suggesting that it is possible to simultaneously target the two proteins due to their similarities in the overall structures and in the binding site shapes. In fact, chemokine receptors have a common molecular architecture, which is conserved within the family of G protein-coupled receptors (GPCRs). As members of this unique family, CCR5 and CXCR4 share a structure similarity (Figure 2, top), such as the presence of seven transmembrane domains. Both receptors consist of 352 amino acids with a high number of proline residues, a C-terminal threonine and serine-rich cytoplasmic region, four extracellular loops rich in cysteines and an N-terminal extracellular domain [6,8]. In particular, overlapping regions of the binding pockets of CCR5 and CXCR4 (Figure 2, bottom) may contribute to allow these two proteins to host the same ligand, although not necessarily in the same binding mode and with an identical affinity.

Due to the marked similarities between the active sites of CCR5 and CXCR4, many ligands could be recognized by both receptors. However, RANTES, also known as chemokine ligand 5 (CCL5), was proven to be the natural ligand selective for CCR5 [11], whereas stromal cell derived factor 1 (SDF1) is the natural ligand of CXCR4 [12]. It is well known that the multi-step HIV entry process is not only mediated by the interaction between the viral gp120 with the cellular receptor CD4, but it also requires the activation of one or two chemokine co-receptors. Among these, CCR5 and CXCR4 exhibit this crucial role. The capability of HIV to use either CCR5 or CXCR4, or both at the same time, determines the viral tropism, since, as already stated. In particular, virus strains utilizing CXCR4 or both co-receptors are associated with a higher incidence of AIDS development [13].

From all these considerations, the challenge to discover new CCR5 and CXCR4 antagonists is of paramount importance to improve the therapeutic armamentarium against HIV infection, and the possibility to develop dual co-receptor antagonists appears particularly appealing.

## 2. CCR5 and CXCR4 Antagonists

### 2.1. Earlier Co-Receptor Antagonists

**Figure 3:**
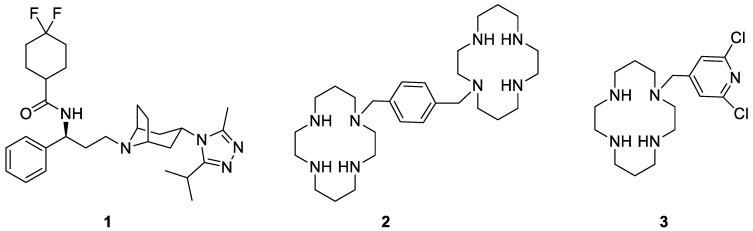
Chemical structures of Maraviroc (**1**), Plerixafor (**2**), and AMD3451 (**3**).

Nowadays, the choice for the clinical use of a CCR5 or CXCR4 antagonists is suggested by the identification of viral strain tropism in patients. A rapid resistance onset represents however a limit, since the inhibition of a single co-receptor drives the virus to shift to the more virulent dual tropic strain [14,15]. Among selective and efficacious CCR5 antagonists, Maraviroc (Figure 3, compound **1**) appeared as the most intriguing compound, being the only one already approved by the Food and Drug Administration (FDA), endowed with a favorable resistance profile. On the other hand, Plerixafor (or AMD-3100) (Figure 3, compound **2**) appeared as the most useful CXCR4 antagonist identified during a research program on anti-HIV agents. Although this compound is clinically used for the mobilization of hematopoietic stem cells in cancer patients, it is not yet approved in the treatment of HIV infection due to its severe side effects [16,17].

In particular, AMD3100 was shown to inhibit HIV-1 entry into the target cell by specifically binding to CXCR4 (and not to any other receptor of either CXC- or CC-chemokines). Previous studies had demonstrated that AMD3100 was very active against a broad range of T-tropic HIV-1 and HIV-2 strains at a low nanomolar concentrations [18], and poorly active against M-tropic strains [19]. Furthermore, AMD3100 was shown to inhibit binding of the CXC-chemokine (SDF-1alpha) to CXCR4 and subsequent signal transduction, thus preventing also the functioning of CXCR4 as CXC-chemokine receptor [20].

In 2004, AMD3451 (Figure 3, compound **3**) was reported as the first dual antagonist of CCR5 and CXCR4 [21]. Although its chemical structure resembles the one of the CXCR4 selective inhibitor AMD3100, it was found to be active also against the CCR5 co-receptor. Its moderate potency did not allow to carry on a proper clinical investigation, but its identification spurred researchers toward the development of more effective dual-tropic CCR5/CXCR4 antagonists [22].

About ten years later it was proven that Maraviroc, initially identified as a selective CCR5 inhibitor, was also an effective therapeutic alternative against dual mixed tropic HIV strains [23]. Antiviral effects of Maraviroc were investigated on U87.CD4 cells expressing wild type or chimeric CCR5 and CXCR4. Furthermore, a direct influence of the drug was observed on the onset of resistance in infected patients [24]. Phase II clinical studies confirmed the susceptibility to Maraviroc of dual mixed tropic viruses, with an activity even higher than that recorded towards pure R5 strains [25]. Nevertheless, Maraviroc is indicated in combination with other antiretroviral agents for the treatment of only CCR5-tropic HIV-1 infection, but not used for dual mixed viruses in the clinic [26].

**Figure 4:**
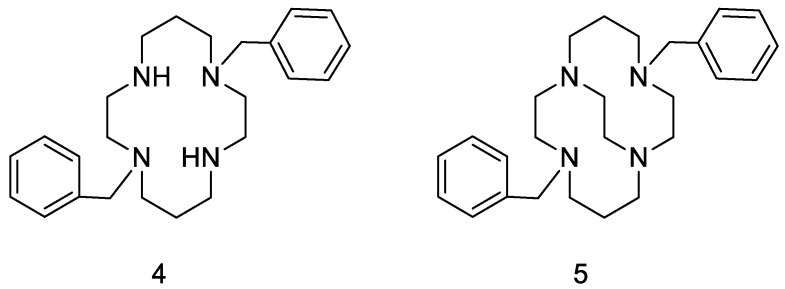
Example of unbridged (**4**) and cross-bridged (**5**) tetraazamacrocycles.

Around the same time, Hubin and coworkers hypothesized that the simultaneous inhibition of CCR5 and CXCR4 by transition metal complexes based on a tetraazamacrocycle could be particularly effective for the treatment of HIV infection (Figure 4) [27,28]. Thermodynamic properties of bridged and unbridged metal complexants were also compared [29]. In a successive report, the same authors developed alternative synthetic routes to include dichloropyridine moieties into the scaffold of these complexants [28].

### 2.2. Peptide-Based Antagonists

**Figure 5:**
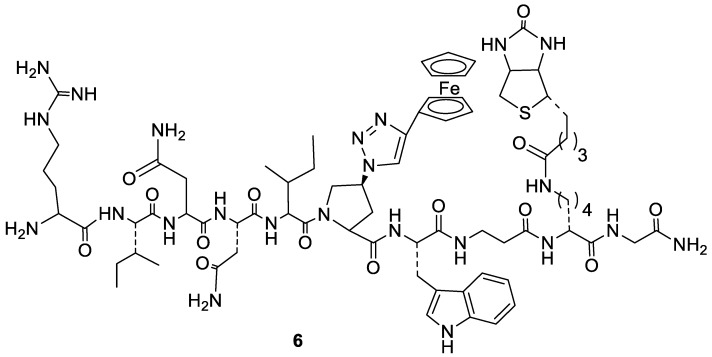
Chemical structure of KR21 (**6**).

In a study focusing on the identification of HIV entry inhibitors, peptide triazoles acting as dual antagonists targeting the interaction of gp120 with CD4 and co-receptors CCR5 and CXCR4 were identified. Eleven out of nineteen gp120 alanine mutants were screened by enzyme-linked immunosorbent assay (ELISA), leading to compound KR21 as the most active agent in direct binding and competition experiments (Figure 5, compound **6**). This peptide corresponds to a conserved region of gp120 overlapping with the CD4 binding site. Amino-acid mutation brought to this compound led to a reduction or even complete loss of inhibitory activity. The overall results of this study suggest that KR21 could represent the starting point for the rationale design of smaller dual antagonists [30].

### 2.3. Diterpene Derivatives

**Figure 6:**
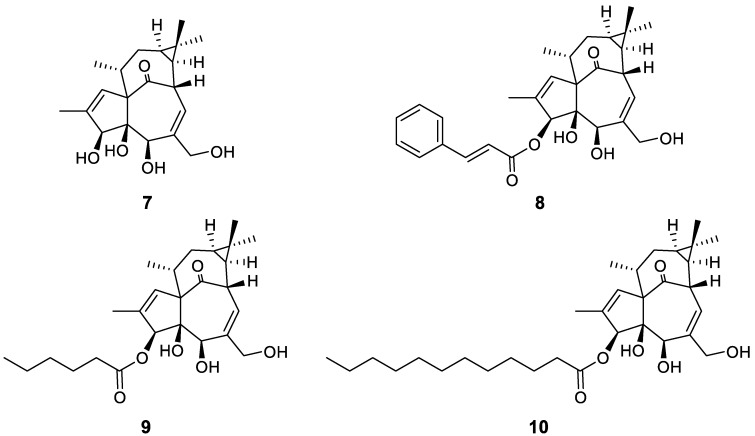
Structure of ingenol **7** and its ester derivatives **8**–**10**.

A new approach to the identification of dual inhibitors was proposed by Gama and coworkers, who described a natural occurring ingenol derivative, a diterpene isolated from the tropical shrub *Euphorbia tirucalli* (Figure 6, compound **7**) [31]. This compound was further chemically manipulated to give active cynnamic, caproic, and lauric esters (Figure 6, compounds **8**–**10**), which showed promising antiviral activity, likely due to the downregulation of membrane receptors CD4, CCR5, and CXCR4. The compounds were tested against dual tropic HIV-1 strains and the EC_50_ values were found to be comparable to those of commonly used antiretroviral drugs. Moreover, these new derivatives, in particular compound **9**, proved to be less toxic than previously discovered ingenol analogs, acting by the modulation of specific protein kinase C isoforms involved in the membrane receptor down-regulation [32].

### 2.4. Pyrazole-Based Antagonists

**Figure 7:**
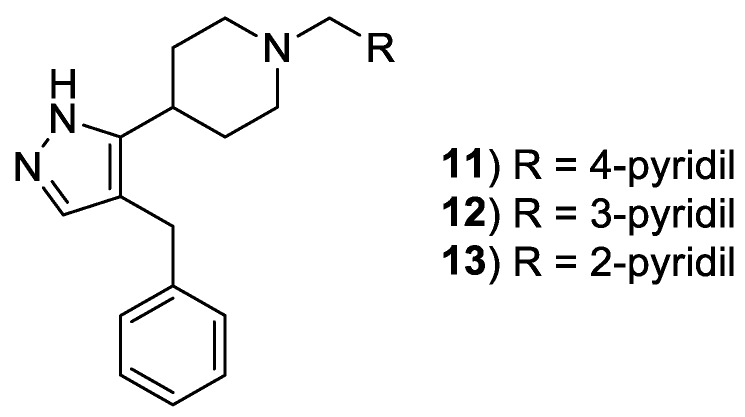
Chemical structure of pyrazolo-piperidine derivatives **11**–**13**.

A composite computational study, based on both virtual screening and statistical approach, led to a series of polyheterocyclic derivatives active on both CCR5 and CXCR4. The core structure was represented by a pyrazolo-piperidine nucleus. The most active derivative (Figure 7, compound **11**), bearing a benzyl group appended to the pyrazole and a 4-pyridinemethyl linked to the piperidine, showed an IC_50_ value of 3.8 µM against a CCR5-utilizing HIV-1 strain and an IC_50_ value of 0.8 µM against a CXCR4-utilizing HIV-1 strain, in MAGI assay. This last consists of a high sensitive competitive in vitro HIV replication method for quantifying viral infectivity. Whereas the benzyl substituent seems necessary for retaining activity, different bonding mode were tolerated for the pyridine ring (Figure 7, compounds **12** and **13**). These compounds showed an IC_50_ value of 17 and 25 µM in the case of the 3-pyridinemethyl derivative and 16 and 5.8 µM in the case of the 2-pyridinemethyl analog against a CCR5- and CXCR4-utilizing HIV-1 strain, respectively. Compound **11** showed also to be active in an assay on Ca^2+^ flux GPCR signaling, therefore allosterically modulating CXCR4. Furthermore, compound **11** showed to be active against HIV-1 reverse-transcriptase enzyme with an IC_50_ value of 9.0 μM. Moreover, this compound did not result toxic in the same MAGI assay, at a concentration as high as 300 µM. All these data suggest that this lead compound is warranted for further development for the identification of more active dual chemokine receptor inhibitors [33].

**Figure 8:**
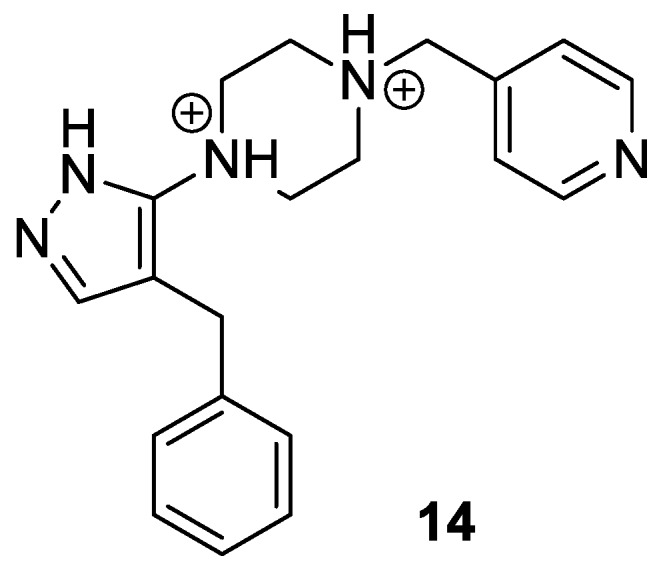
Chemical structure of piperidine derivative **14**.

In a successive computational study, the dynamics of the binding between **11** and both CCR5 and CXCR4 were in depth investigated [34]. The three aromatic rings involved in π-stacking and a positively charged hydrogen bond donor of a piperidine ring were demonstrated to be the main responsible features for the interaction. The replacement of the piperidine ring with a piperazine, leading to a double protonated species interacting with the negatively charged glutamates and aspartates within the active site, was planned in order to strengthen the protein-ligand interaction. Accordingly, compound **14** (Figure 8) was synthesized and demonstrated to have a more favorable interaction compared to **11**, after being docked into the active site of both co-receptors [34]. These results suggested that further insights into the molecular dynamics of such compounds and CCR5/CXCR4 could lead to the identification of more effective dual antagonists.

### 2.5. The Suramin Analog NF279

**Figure 9:**
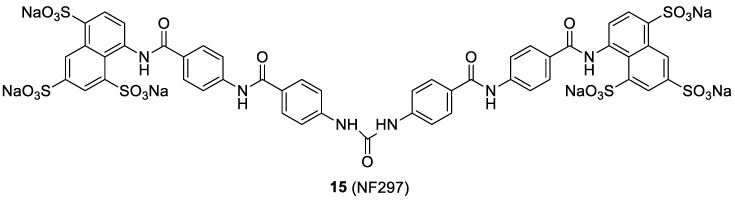
Chemical structure of compound **15**.

Inhibition by selective antagonists of P2X1R, a receptor involved in the HIV-1 fusion and replication, could represent an alternative strategy to contrast the viral infection. Compound **15**, also known as NF279 (Figure 9), an analog of the anti-parasite drug suramin, was initially found to be as a selective P2X1 receptor antagonist, and showed a noteworthy antiviral activity [35]. Further studies proved that HIV-1 entry inhibitory activity of compound **15** was not related to a direct interaction with P2X1R, but rather to the capability of this compound to act as dual CCR5/CXCR4 co-receptor antagonist. This feature was assessed by the suppression of cellular calcium responses CCR5/CXCR4 mediated. Thus, NF279 could represent the lead of a new class of dual-coreceptor inhibitors [36].

### 2.6. The Cumarin-Based Ligand GUT-70

**Figure 10:**
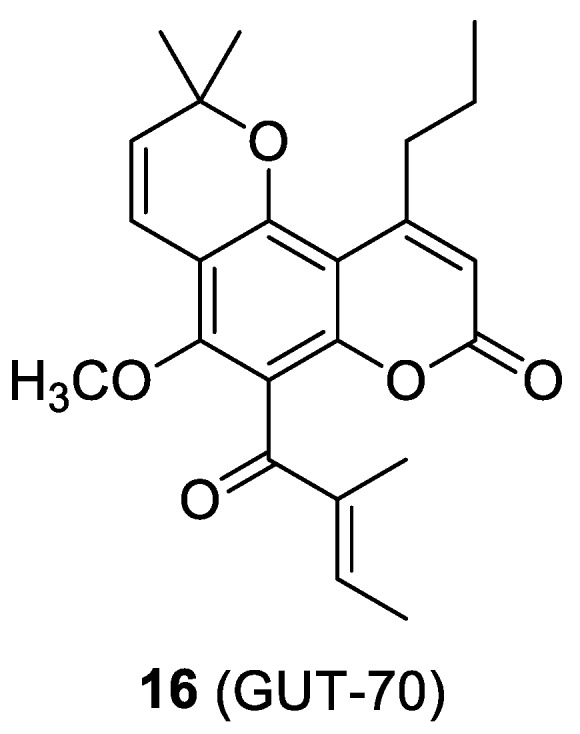
Chemical structure of compound **16**.

It is known that several derivatives of the natural product coumarin are endowed with several pharmacological properties, including anti-viral activity [37]. Compound **16**, a tricyclic coumarin also known as GUT-70 (Figure 10), was demonstrated to be able to reduce cell membrane fluidity by a fluorescent depolarization study conducted on MOLT-4 and PM1-CCR5 T cell lines. This property would suggest its potential use against the HIV-1 entry. Furthermore, GUT-70 is capable of down-regulating the expression of CD4, CCR5, and CXCR4 receptors on the cell surface in a dose-dependent manner, therefore representing a starting point for the development of novel tools against HIV-1 infection [38]. However, this compound showed an unfavorable toxicity profile, due to a noteworthy cytotoxicity [39].

### 2.7. Penicillixanthone A

**Figure 11:**
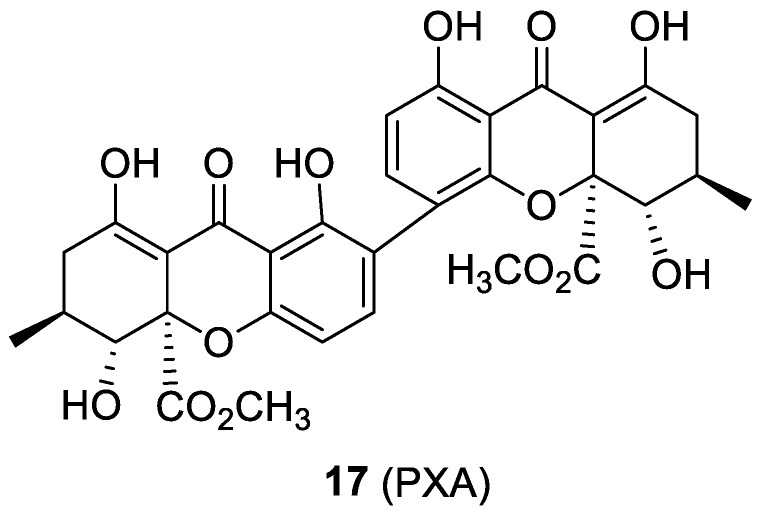
Chemical structure of compound **17**.

It is well known that HIV fusion can be humped by a peptide portion of gp41 (HR2 domain). An antiviral effect has been demonstrated when this peptide portion was conjugated to cell membrane proteins. In particular, a 34 amino acid peptide from HR2 (C34, corresponding to amino acids 628–661 in HxB2) [40,41] conjugated to the amino termini of CCR5 or CXCR4 exhibited potent and broad inhibition against several HIV-1 strains [42]. Potent anti-HIV-1 activity was showed by penicillixanthone A (PXA, Figure 11, compound **17**), a natural xanthone dimer isolated from the fungus *Aspergillus fumigatus*. The actual mechanism of action of PXA was inferred by molecular modeling studies, which suggested a putative interaction binding mode with both CCR5 and CXCR4 receptors. The IC_50_ values calculated by in vitro studies were 0.36 and 0.26 μM against CCR5- and CXCR4-tropic HIV-1 strains, respectively. PXA showed only a moderate cytotoxicity evaluated by a colorimetric XTT assay against TZM-bl cells, with an IC_50_ of 20.6 μM. This compound could then represent a lead for the development of HIV-1 dual co-receptor antagonists [43].

Considering all the above remarks, the identification of dual CCR5/CXCR4 co-receptor antagonists is still an attractive field of anti-HIV research. In fact, the progression of HIV infection is associated with the skill of the virus to switch from one co-receptor to another. Although our understanding of the viral resistance mechanism is not yet complete, the alternative therapeutic option based on the inhibition of membrane co-receptors sounds still appealing. Such an approach could gain particular importance in the case of anti-HIV treatment failure with classic HAART therapy. After the identification of effective antagonists, the next step could reside in the choice of a specific therapy based on the use of selective or dual agents, or even a combination of both types.

## 3. Conclusions

Successful applications of ligand-based models and recent insights on the mechanism of HIV replication prompted the research of a new strategy aimed at preventing viral adhesion and spread. A promising approach is based on the employment of agents able to antagonize the interaction of viral proteins with the host cell membrane receptor CD4 and co-receptors CCR5 and CXCR4. The viral tropism is in fact thoroughly influenced by the expression of one or both co-receptors. The identification of selective co-receptor antagonists could be particularly effective in preventing viral infection. However, the capability of the virus to switch tropism could reduce drug effectiveness. In this context, the discovery of dual CCR5 and CXCR4 could lead to even more appealing anti-viral agents, able to prevent the replication of the more virulent dual-tropic HIV strains. The studies so far conducted for the identification of dual inhibitors have led to interesting candidates to be further developed, even if several of the identified compounds suffer from an inadequate safety and pharmacokinetics profile. Most of the analyzed papers deal with in vitro and in silico evaluations, which often do not reflect therapeutic potentials for a suitable clinical application. Rationale multidisciplinary efforts combining computational and biological techniques are then required for the design of leads to be developed toward clinically useful potent antiviral agents.
